# 

**Published:** 1868-08

**Authors:** 


### CONTENTS OF HALL'S JOURNAL OF HEALTH FOR AUGUST.

Is Consumption Contagious? .......... 169
Table Manners, .......... 170
Tomatoes, .......... 172
Ice Water, .......... 173
Regimen, .......... 174
Sleeping, .......... 175
Tobacco-Users, .......... 177
Cellars, .......... 178
Spitting Blood, .......... 179
Small Bed-chambers, .......... 180
Each the Best, .......... 181
Eight to Sixteen, .......... 183
Drinking Water, .......... 184
Drunken Women, .......... 185
Metamorphoses, .......... 187
Catarrh, .......... 189
Living Yet, .......... 190

